# Study on the preventive effect of ramelteon on the onset of sleep disorder after general anesthesia in patients with autism spectrum disorder: A study protocol

**DOI:** 10.1097/MD.0000000000022826

**Published:** 2020-10-23

**Authors:** Terumi Ayuse, Yu Ozaki-Honda, Shinji Kurata, Gaku Mishima, Kensuke Kiriishi, Nobuaki Magata, Hanako Kawasaki, Kaori Yamaguchi-Komeyama, Naomi Tanoue, Takao Ayuse

**Affiliations:** a Department of Special Care Dentistry; b Department of Dental Anesthesiology, Nagasaki University Hospital; c Division of Clinical Physiology, Department of Translational Medical Sciences, Nagasaki University Graduate School of Biomedical Sciences, Nagasaki, Japan.

**Keywords:** autism spectrum disorder, general anesthesia, Insomnia, sleep disorder

## Abstract

**Background:**

The persistent loss of consciousness caused by general anesthesia without the existence of repeated 90-minute cycles of non-REM and REM sleep might significantly disturb and suppress the cycle of normal physiological sleep in postoperative periods after general anesthesia. Patients with autism spectrum disorders (ASD) with existing circadian rhythm disorder are reported to rapidly deteriorate due to acute sleep disorder during the perioperative period after general anesthesia.

A melatonin receptor agonist, ramelteon (Rozerem), which is a sleep cycle regulator, is used as a therapeutic drug for patients with sleep disorders, but there are no studies on the prevention of postoperative sleep disorder after general anesthesia.

In this study, we investigate whether prophylactic administration of a sleep-inducing substance, a melatonin receptor agonist, is effective against sleep disorder after general anesthesia in patients with ASD.

**Methods/design:**

This study is intended for patients with ASD aged 12 years and above who undergo treatment at Nagasaki University Hospital, Isahaya General Hospital Dentistry, and Sasebo City General Medical Center Dentistry and undergo dental treatment under general anesthesia. A melatonin receptor agonist (Rozerem) will be taken 7 days prior and 7 days postsurgery in patients diagnosed with insomnia. A randomized comparison will be made between 2 groups: an experimental group that is additionally administered Rozerem and a control group.

The primary endpoint is the incidence of NREM-REM sleep disorders that occur within 3 to 5 days after general anesthesia. The secondary endpoint is the incidence of circadian rhythm sleep disorders (rate of occurrence of sleep-retardation syndrome with drowsiness and strong fatigue).

**Discussion:**

Postoperative sleep disorders after general anesthesia has been reported in patients with ASD; however, effective preventive pharmacological treatments have not been established. A sleep cycle regulator, ramelteon (Rozerem), is used as a therapeutic drug for patients with sleep disorders by decreasing the difficulty of falling asleep in insomnia. If sleep disorder can be prevented after the administration of general anesthesia in patients with ASD, we can support social participation while maintaining their quality of life.

Trial registration: The study was registered with the jRCT1071200030.

## 1 Introduction

The persistent loss of consciousness (non-REM sleep only) caused by general anesthesia without the existence of repeated 90-minute cycles of non-REM sleep and REM sleep might significantly disturb and suppress the cycle of normal physiological sleep in the postoperative period after general anesthesia. As a result, it has been suggested that sleep disorders may be caused mainly by circadian rhythm disorders such as increase in recoil of REM sleep even in the perioperative period.

Although various complications of these sleep disorders have been reported, it has become clear that they have a great influence on the mental and cognitive functions. It has been reported that, patients with autism spectrum disorders, with existing circadian rhythm disorder rapidly deteriorate due to acute sleep disorder during the perioperative period after general anesthesia.^[1–5]^ Furthermore, when sleep disorders occur after the administration of general anesthesia in the elderly with mildly deteriorated brain function, not only does acute postoperative delirium develop within a few days of the operation but also subacute postoperative operation occurs within a few weeks. The risk of cognitive impairment has also been pointed out. It has been reported that the risk of developing sleep disorders after general anesthesia is related to factors such as long-term anesthesia, old age, and the presence of sleep disorders before surgery. The risk of abrupt deterioration of cognitive behavioral function due to sleep disorders during the perioperative period has also been reported, and thus, active prevention of postoperative delirium is important for people with disabilities such as autism spectrum disorders (ASD).

However, there is no consensus on how the sleep disorders that occur during the perioperative period after general anesthesia affect the mental and cognitive functions of patients with disabilities like ASD. To date, no effective method of preventing the onset has been established.

The melatonin receptor agonist Rozerem, which is a sleep cycle regulator, is used as a therapeutic drug for patients with sleep disorders, but no research has been reported for preventing postoperative sleep disorder after general anesthesia. Studies have been conducted to prevent delirium after anesthesia by prophylactic administration of the melatonin receptor agonist (Rozerem) in children undergoing general anesthesia,^[6–9]^ but prior to the administration of the melatonin receptor agonist. In addition to studies that examined postoperative pain level and sleep quality, prevention of sleep disorders after anesthesia in people with disabilities such as ASD secondarily reduces postoperative delirium. No intervention studies have examined whether to do so.

In this study, we investigate whether the prophylactic administration of a sleep-inducing substance, a melatonin receptor agonist, is effective against sleep disorder after general anesthesia in patients with ASD.

## 2 Methods/design

### 2.1 Study design

The present study was designed in accordance with the Standard Protocol Items: Recommendations for Interventional Trials and Consolidated Standard of Reporting Trials 2010 guidelines.^[10,11]^ This was a randomized, controlled study for patients with ASD scheduled for dental treatment under general anesthesia.

Patients with ASD at Nagasaki University Hospital, Isahaya General Hospital Dentistry, and Sasebo City General Medical Center Dentistry over the age of 12 years scheduled for dental treatment under general anesthesia will be targeted and enrolled.

**Figure 1:**
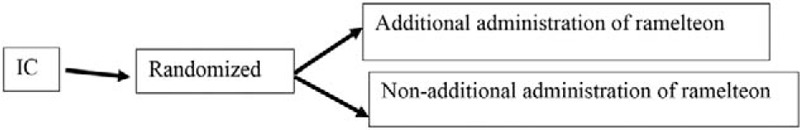
A randomized comparison will be made between the 2 groups: an experimental group that is additionally administered with Rozerem and a control group (ratio 1:1).

A melatonin receptor agonist (Rozerem) was taken 7 days prior and 7 days after dental treatment in a patient who had been diagnosed with insomnia before admission and is currently taking insomnia medication. A randomized comparison will be made between the 2 groups: an experimental group that is additionally administered with Rozerem and a control group (Fig. 1). We randomly assign target patients who meet the registration requirements.

The Clinical Research Review Board in Nagasaki University approved the study and study protocol. The study was conducted at Nagasaki University Hospital in Japan. The study is registered on the jRCT. The study was conducted in accordance with the principles of the Declaration of Helsinki and the established best clinical practices of Japan. This is an exploratory study aimed at collecting information for conducting a verification study. Therefore, the number of cases is set based on the feasibility in our hospital.

### 2.2 Participant recruitment

Targeting ASD patients undergoing planned dental treatment under general anesthesia at Nagasaki University Hospital, Isahaya General Hospital Dentistry, Sasebo City General Medical Center Dentistry. Patients who have already been treated with drugs other than Rozerem for insomnia, and have no history of sleep disorders in the past, but before admission to the hospital, the insomnia scale of 4 to 5 points for “insomnia Patients with ”suspect“ or ”high probability of insomnia“ with a score of 6 or more are targeted.

### 2.3 Inclusion criteria

Patients who meet all parameters of the following criteria will be enrolled:

1. Age: People with disabilities who are 12 years or older at the time of obtaining consent.
2. Patients who have already been diagnosed with insomnia and have been given oral treatment with drugs other than Rozerem.
3. Patients who have not been diagnosed with sleep disorders in the past, but are ”probable insomnia“ with a score of 4 to 5 on the Athens insomnia scale or ”probable insomnia“ with a score of 6 or more before admission to the hospital.
4. Patients without serious systemic complications (ASA PS I-II).
5. Gender: Any.
6. Hospitalization/Outpatient: Hospitalization only.
7. Patients who, after receiving a sufficient explanation before participating in this study, have a thorough understanding and have provided informed consent or have the informed consent of their parents.

### 2.4 Exclusion criteria

Patients who meet at least 1 of the following are excluded as subjects.

1. Patients who have already taken Rozerem
2. Breast-feeding woman: Breast-feeding woman
3. Patients with serious complications (ASA PSIII or higher)
4. Patients with a history of hypersensitivity to Rozerem
5. Patients with advanced liver dysfunction
6. Patients receiving fluvoxamine maleate
7. Other patients who the investigator determined to be unsuitable for study

#### 2.4.1 Sample size

The sample size is set at 45 patients in 1 group with ASD, and 90 patients are set in total. This research is an exploratory research aiming to collect information for conducting verification research. Therefore, the number of cases is set based on the feasibility.

A study, with 80 patients, investigating the effect of ramelteon on the prevention of postoperative delirium in older patients undergoing orthopedic surgery indicated 9% delirium incidence during the 2 days following surgery.^[12]^ Referring to the results of this study, it is expected that the effect of melatonin agonists will be similar in patients with ASD with existing sleep disorders. Assuming that the incidence of postoperative sleep disorders is 50% in the Rozerem non-additional administration group, it will be about 80 cases when calculated with the ratio of α = 0.05, power = 0.8. Statistical processing is possible in about 90 cases, while considering a 10% dropout. Therefore, in patients with ASD, we set the sample size at around 45 cases in each group, and conducted a study with a target sample size of 90 cases.

### 2.5 Study protocol

Patients who received study consent and were additionally administered a melatonin receptor agonist called ramelteon (Rozerem ) as an additional drug were assigned to the experimental group 8 days before general anesthesia and 7 days after surgery. They are orally administered 8 mg Rozerem once a day for 14 days at bedtime with an insomnia drug that is a carry-on drug. Patients assigned to the target group who do not additionally administer Rozerem in addition to the insomnia drug will be orally administered only the insomnia drug for 14 consecutive days which will be 7 days before general anesthesia and 7 days after the operation.

### 2.6 Adverse events

While the study is conducted, an allergic reaction may occur in the group administered with Rozerem and care should be taken not to burden the research subjects.

### 2.7 Outcome

The primary endpoint is the incidence of NREM-REM sleep disorders that occur within 3 to 5 days after general anesthesia.

The secondary endpoint is the occurrence rate of circadian rhythm sleep disorder (incidence rate of sleep phase delay syndrome with drowsiness and strong fatigue).

### 2.8 Efficacy

We evaluated and analyzed the efficacy of the investigational device on several parameters, which mainly included the difference in the incidence of sleep disorders between the group administered the additional dose of Rozerem and the group without the additional administration of Rozerem.

### 2.9 Safety

The safety evaluation indices of this clinical trial are as follows: adverse events are any undesired or unintended signs (including abnormal laboratory values, abnormal vital signs), symptoms, or illnesses that occur between the start of drug use and the end of the last observational study. It does not matter whether the study has a causal relationship. Symptoms and diseases occurring before the use of drug are treated as complications and not adverse events. However, if there are complications after commencement of the drug, they will be treated as adverse events, and the day on which the deterioration is confirmed will be considered as the date of the occurrence of the adverse events.

### 2.10 Data collection and management

The assignment table and input table used in this study were created with Research Electronic Data Capture (REDCap). The study will be conducted after allocating the registered patients, and the data of all items in the medical record collected in the study were assigned to the researcher who was assigned the ID entered by the physician, co-doctor, and a co-worker. The Principal Investigator or Co-Researcher approved the input observation/inspection/evaluation data of each research subject immediately after confirming the content. For the data entered in the case report, the Principal Investigator and the Clinical Research Center Data Management staff performed a visual check and a logical check. Consequent to each check, if there are any problems or doubts in the data, the principal investigator, or the research coordinator is contacted. The case is fixed by performing a data lock on the case when the issue has been resolved, and any modifications have been completed. If there is an error that needs to be corrected after the case is locked, the data management staff is responsible for overseeing this process.

In this study, the monitoring will be carried out in accordance with the research plan and monitoring procedures to ensure that the research is being conducted properly.

### 2.11 Statistical analysis

Since this study is exploratory, we estimated an incidence rate for each study parameter and then used this information to calculate the necessary sample size for the verification study to achieve statistical significance.

Specifically, the differences in the incidence of sleep disorders between the group administered the additional dose of Rozerem and the group not administered the additional dose of Rozerem will be analyzed by Fishers exact test. For example, the analysis is performed assuming that 5 out of 30 cases of the intervention group (group with the administration of the additional dose) and 15 out of 30 cases of the group with the additional dose of Rozerem are not performed. We would test the null hypothesis: ”There is no difference in the incidence of sleep disorders between the group with the additional dose of Rozerem and the group without the additional dose of Rozerem".

## 3 Discussion

This study is an intervention study to investigate whether the development of sleep disorder after general anesthesia in patients with ASD can be reduced by the prophylactic administration of a melatonin receptor agonist named ramelteon (Rozerem). The risk of complications such as postoperative cognitive dysfunction due to sleep disorders after general anesthesia has been reported in patients with ASD, however effective prevention methods have not been established.

The sleep cycle regulator, melatonin receptor agonist (Rozerem), is used as a therapeutic drug for patients with sleep disorders in the hope of improving the difficulty of falling asleep in insomnia, but sleep disorders after general anesthesia have not been tested for prevention.

It has been indicated that the increased amount of daytime sleep is considered to reduce sleep efficiency, REM sleep, and slow-wave sleep.^[13]^ We suppose that the existence of daytime insomnia symptoms due to reduced total daytime sleep are commonly experienced with ASD^[14,15]^ and might be a major factor to potentiate sleep cycle disturbance after general anesthesia.

It is emerging that general anesthesia strongly affects postoperative sleep architecture and/or circadian rhythm.^[16]^ However, much remains unclear through what mechanism general anesthesia affects sleep architecture or the circadian rhythm at both the cellular level of the central nervous system and from a behavioral perspective. To elucidate the clinical significance of postoperative sleep disorder, it is necessary to conduct both molecular biology investigations as well as behavioral studies. It may also be necessary to develop a standardized protocol which considers the potential for postoperative sleep disorder following general anesthesia.

In terms of general anesthesia, unlike healthy adults, individuals with ASD often encounter communication difficulties preoperatively. Patients may experience stress not only as a result of being sedated under general anesthesia, but also due to what may seem like a daunting visit to the hospital. Patients with ASD commonly present with sleep disorders have been observed even under normal living conditions, and there are studies that have indicated the association between sleep disorder and self-harming behavior.^[17]^

In the present study, the assessment of sleep began at home, 7 days before general anesthesia. These everyday sleep patterns, specific to the individuals with disability, served as preoperative control values. Thus, even if the patients’ sleep architecture was already modulated compared to other patients who do not display underlying mental disorders, the patient-specific preoperative values can be used to compare the changes in sleep architecture after general anesthesia. If some type of sleep disorder were to occur after general anesthesia, even for a short duration, the patient would be at risk of developing extremely serious complications when combined with a pre-existing sleep disorder.

It would be important to consider that sleep disorder has a strong relationship between sleep quality and oral medication, such as anti-epileptic and/or anti-psychotic drugs. Patients with ASD associated with intellectual disability often have a pre-existing sleep disorder.^[1–4,18–20]^ It has been suggested that insomnia symptoms of sleep problems, such as increased sleep onset latency, increased wake after sleep onset episodes, and reduced total sleep time, are commonly experienced with ASD.^[14,15]^ Therefore, we consider that daily medication with these drugs for maintenance of mental condition might have a major influence on sleep quality. It is reasonable to estimate that there is a significant risk factor for sleep disorder in patients with ASD or mental retardation.

For minor surgical or dental procedures under general anesthesia in individuals with disabilities, ascertaining the quality of everyday sleep is necessary, in addition to evaluating systemic complications and the patients general condition. In our previous study, we have evaluated sleep architecture perioperative periods using a mat type sleep monitoring device. To achieve increased measurement accuracy for a long duration in perioperative periods, it would be necessary to investigate the characteristics of the patients sleep architecture by screening, using an compact and lightweight wrist watch type sleep meter that might be acceptable to the these patients. If a patient presents with a significant preoperative sleep disorder, treatment with sleep aids such as hypnotics and melatonin should be considered upon consultation with the attending physician.

If there is existence of insomnia before general anesthesia, it is necessary to normalize sleep with various sleep regulators. In addition, it would be appropriate to use sedative drugs to prevent postoperative acute agitation after general anesthesia which may lead to secondary postoperative sleep disturbance.^[21]^ We suppose that preoperative treatment for insomnia by administration of such a medicine might be clinically relevant.

If we can reduce and prevent sleep disorders after general anesthesia in mentally disabled patients, especially in patients with ASD, we will be able to discharge them earlier without complications, and not only save medical costs but also enable social participation while maintaining the quality of life for patients with ASD.

## Acknowledgments

The authors would like to thank our colleagues and staff at the Dental Anesthesiology, Department of Nagasaki University Hospital for their support.

## Author contributions

Terumi Ayuse, Yu Ozaki-Honda, and Takao Ayuse are responsible for conceiving and designing the trial, planning data analysis, drafting the manuscript, and approving the final manuscript. TA, GM, KK, YO, NM, HK, KY, NT will participate in data collection and will oversee the recruitment and treatment of patients. All authors will have access to the interim results as well as the capacity to discuss, revise, and approve the final manuscript.

**Conceptualization:** Terumi Ayuse, Yu Ozaki-Honda, Takao Ayuse.

**Data curation:** Shinji Kurata, Gaku Mishima, Kensuke Kiriishi, Nobuaki Magata, Hanako Kawasaki.

**Methodology:** Terumi Ayuse, Shinji Kurata, Gaku Mishima, Kensuke Kiriishi, Nobuaki Magata, Hanako Kawasaki, Kaori Yamaguchi-Komeyama, Naomi Tanoue.

**Writing – original draft:** Terumi Ayuse.

**Writing – review & editing:** Yu Ozaki-Honda, Takao Ayuse.
